# An evaluation of isolation by distance and isolation by resistance on genetic structure of the Persian squirrel (*Sciurus anomalus*) in the Zagros forests of Iran

**DOI:** 10.1002/ece3.10225

**Published:** 2023-07-04

**Authors:** Marzieh Asadi Aghbolaghi, Nusha Keyghobadi, Zeinab Azarakhsh, Marzieh Dadizadeh, Shahab Asadi Aghbolaghi, Navid Zamani

**Affiliations:** ^1^ Department of Biodiversity and Ecosystem Management, Environmental Sciences Research Institute Shahid Beheshti University Tehran Iran; ^2^ Department of Biology The University of Western Ontario London Canada; ^3^ Center of Remote Sensing and GIS Research, Faculty of Earth Sciences Shahid Beheshti University Tehran Iran; ^4^ Department of Education of Chaharmahal and Bakhtiari Province (Ministry of Education) Shahrekord Iran; ^5^ Department of Environmental Science, Faculty of Natural Resource University of Kurdistan Sanandaj Iran

**Keywords:** IBD and IBR, landscape genetic, *Sciurus anomalus*, Zagros forest

## Abstract

For the conservation of wild species, it is important to understand how landscape change and land management can affect gene flow and movement. Landscape genetic analyses provide a powerful approach to infer effects of various landscape factors on gene flow, thereby informing conservation actions. The Persian squirrel is a keystone species in the woodlands and oak forests of Western Asia, where it has experienced recent habitat loss and fragmentation. We conducted landscape genetic analyses of individuals sampled in the northern Zagros Mountains of Iran (provinces of Kurdistan, Kermanshah, and Ilam), focusing on the evaluation of isolation by distance (IBD) and isolation by resistance (IBR), using 16 microsatellite markers. The roles of geographical distance and landscape features including roads, rivers, developed areas, farming and agriculture, forests, lakes, plantation forests, rangelands, shrublands, and rocky areas of varying canopy cover, and swamp margins on genetic structure were quantified using individual‐based approaches and resistance surface modeling. We found a significant pattern of IBD but only weak support for an effect of forest cover on genetic structure and gene flow. It seems that geographical distance is an important factor limiting the dispersal of the Persian squirrel in this region. The results of the current study inform ongoing conservation programs for the Persian squirrel in the Zagros oak forest.

## Graphical abstract

The Persian squirrel, *Sciurus anomalus*, has a high conservation value as a keystone species in the Middle East's woodlands and oak forests. We focused on analysis of IBD and IBR using an individual‐based approach, to identify features that may potentially explain contemporary genetic structure in the northern Zagros region. Our results revealed geographical distance as the main factor that structures contemporary genetic variation among Persian squirrels in the North Zagros.

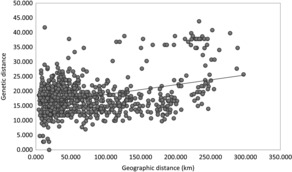

## 1 INTRODUCTION

Natural populations occur in a landscape mosaic where dispersal patterns among local populations and population structure are affected by environmental features that differentially limit or promote movements of individuals and their genes (Gharehaghaji et al., 2017; Mapelli et al., 2012). Landscape genetics provides a means to empirically assess and quantify how such features influence gene flow and genetic connectivity (Manel et al., 2003; Storfer et al., 2007). Central to many landscape genetic studies is the concept of isolation by resistance (IBR), where different landscape elements reduce movement and gene flow to varying degrees (i.e., provide differential resistance; McRae, 2006). Typically, one or more specific IBR hypotheses, postulating relative resistance values for different landscape features, are compared with each other and to the hypothesis of isolation by distance (IBD; Wright, 1943). IBD is the model for describing spatial genetic patterns among populations under limited movement, where the degree of genetic differentiation is solely a function of the Euclidean distance between individuals or populations (Ishida, 2009; Rohlf & Schnell, 1971). The IBR framework can improve understanding of how a broad range of landscape features and characteristics simultaneously influence gene flow and movement, ultimately allowing researchers to predict both ecological and evolutionary consequences of landscape change. By investigating the roles of isolation by distance (IBD) and isolation by resistance (IBR) in determining genetic structure, we can gain a more complete understanding of the effects of spatial distance and habitat heterogeneity on species distribution, abundance, and movement (Kunz et al., 2022; McRae, 2006).

An understanding of animal movement and genetic connectivity is important, in turn, for population management and landscape planning (Russo et al., 2016). Tree squirrels (Sciuridae) are highly mobile animals that depend mainly on forest habitat, preferably deciduous forest, to survive and may not easily move through non‐forested environments (Bakker & Van Vuren, 2004; Bowman & Fahrig, 2002; Bridgman et al., 2012; de Abreu‐Jr et al., 2020; Stevenson et al., 2013). The distribution of tree squirrels is strongly affected by the extent and spatial patterns of forests, and the evolution of tree squirrels has been shaped by the plant resources on which they depend for food, nest sites, and protection from predators (Di Febbraro et al., 2019; Koprowski, 1991; Steele, 2008; Steele & Koprowski, 2001). At the same time, these small rodents provide important ecosystem services as pollinators and seed dispersers and their activities support forest regeneration (Flores‐Manzanero et al., 2019; Russo et al., 2016). Therefore, understanding tree squirrel movement and genetic connectivity can inform landscape management to promote conservation.

The Persian squirrel or Caucasian squirrel (*Sciurus anomalus*; Güldenstädt, 1785) is a tree squirrel occurring in the Iran‐Anatolian region and the east coast of the Mediterranean Sea. The distribution of Persian squirrels is known to be affected by the extent and spatial patterns of forested areas (Demirtaş, 2022; Etemad, 1978; İbiş et al., 2022; Karami et al., 2016; Masseti, 2005; Oshida et al., 2009). Specifically, this species occurs in forested areas dominated by oak, olive, pine, and pistachio (Amr et al., 2006; Arslan et al., 2008; Harrison & Bates, 1991; Hatt, 1959; Khalyani & Mayer, 2013; Koprowski et al., 2016; Malekian & Sadeghi, 2020; Osborn, 1964; Özkurt et al., 1999; Thorington Jr et al., 2012; Yiğit et al., 2006). The species tends to build nests in cavities of oak trees and derives nutrition from the seeds of oak trees; in return, the Persian squirrel buries oak seeds into the soil, and this food caching activity contributes to seed germination and forest regeneration (Karami et al., 2016). As a result, the Persian squirrel has been described as a keystone species in the forests of these regions (Karami et al., 2016). The species tends to select nesting areas with higher protection from predators and greater food availability, and specific factors identified as being important to the choice of nesting tree include crown canopy cover, number of trees in the plot, and distance to the nearest tree (Khalili et al., 2016). Dispersal rates and distances in the Persian squirrel have not been directly quantified, but based on mean natal dispersal distances of approximately 1 km (with a maximum of 4 km) in the closely related Eurasian red squirrel (Wauters et al., 2010), the Persian squirrel may have a similar, moderate dispersal ability.

While the Persian squirrel is listed as “Least Concern” (LC) by the IUCN, the current population trend is decreasing (Yiğit et al., 2016). Habitat loss and fragmentation are considered important threats responsible for population decline of the Persian squirrel, especially in Lebanon, Syria, and Iran (Aidek et al., 2022; Harrison et al., 2006; Yiğit et al., 2008; Zannetos et al., 2022). Destruction of Zagros forests in Iran seems to be the most serious threatening factor (Karami et al., 2016), while climate change also has the potential to negatively impact the habitat of the Persian squirrel through the 21st century (Harrison et al., 2006). Species distribution models under climate change projections for the year 2050 revealed a significant reduction in the area of suitable habitats for this species in Iran, potentially as a result of reduced winter precipitation (Malekian & Sadeghi, 2020). Furthermore, loss of suitable habitats under climate change is predicted to be most pronounced in the northern Zagros, where some of the best habitats currently occur (Malekian & Sadeghi, 2020). In addition, many Persian squirrels are captured every year to be sold in markets as pets (Kolosov et al., 1965; Vereshchagin & Vereshchagin, 1967). Currently, there are not any valid estimates of the population size of this species in Iran. It is necessary to obtain more information about the ecology, taxonomy, habitat, and main threats to this species, so that conservation interventions can be implemented before it declines further. Analyses of genetic structure and landscape genetics that can reveal the current genetic status of the populations, and enhance our knowledge of which environmental factors most affect gene flow, can be useful to inform conservation strategies for the species (Darinot et al., 2021).

Based on phylogenetic and biogeographical studies, the diversification of the Persian squirrel across its current range can potentially be attributed to Pleistocene climatic fluctuations, with isolation and differentiation having occurred in refugial forest areas during glacial periods (Asadi Aghbolaghi et al., 2019, 2020). Persian squirrel populations are divided into at least two main lineages across its whole distribution area, spanning the Greek islands and Mediterranean Sea to the Zagros forest in the Middle East (Asadi Aghbolaghi et al., 2019; İbiş et al., 2022). The two lineages comprise at least five genetically distinct groups, some of which are separated by large geographical distances. In the Zagros forest, two distinct genetic groups were identified: the first group (G4) comprises samples from two distinct areas, the Levant countries in the eastern coast of the Mediterranean Sea (e.g., Syria, Lebanon, Jordan) and the northern part of the Zagros Mountain range (Turkey, Iraq and Iran), while the second group (G5) occurs in the southern Zagros in Iran, which has drier and warmer habitat conditions compared with the northern Zagros (Asadi Aghbolaghi et al., 2019, 2020).

In the current study, we tested the impact of landscape variation on gene flow in the Persian squirrel in the northern Zagros population (G4 in Asadi Aghbolaghi et al., 2019) in Iran (Kurdistan, Kermanshah, and Ilam provinces) using a landscape genetics framework. We hypothesized that forest cover, which provides critical resources and habitat for the species, would facilitate gene flow. Conversely, we hypothesized that non‐forested areas, particularly agriculture, urban and developed areas, and roads, where there might be higher risk of contact with humans and elevated risk of mortality, as well as rivers and lakes which might be impassable barriers, would all restrict gene flow (Bauder, Cervantes, et al., 2021; Blanchong et al., 2008; Locher et al., 2015; Miller et al., 2020; Robinson et al., 2012). We also hypothesized that geographical distance would restrict gene flow because of spatially limited dispersal. We used microsatellite genotyping (Hale, Bevan, & Wolff, 2001; Hale, Lurz, et al., 2001) and an individual‐based approach to estimate IBD and landscape resistance (IBR) of these multiple landscape features. Given the high rates of landscape change in this area, particularly a high level of forest destruction and fragmentation, understanding the role of IBR and IBD in the species can potentially inform better management and conservation plans.

## 2 METHODS

### 2.1 Study site and sampling

**FIGURE 1:**
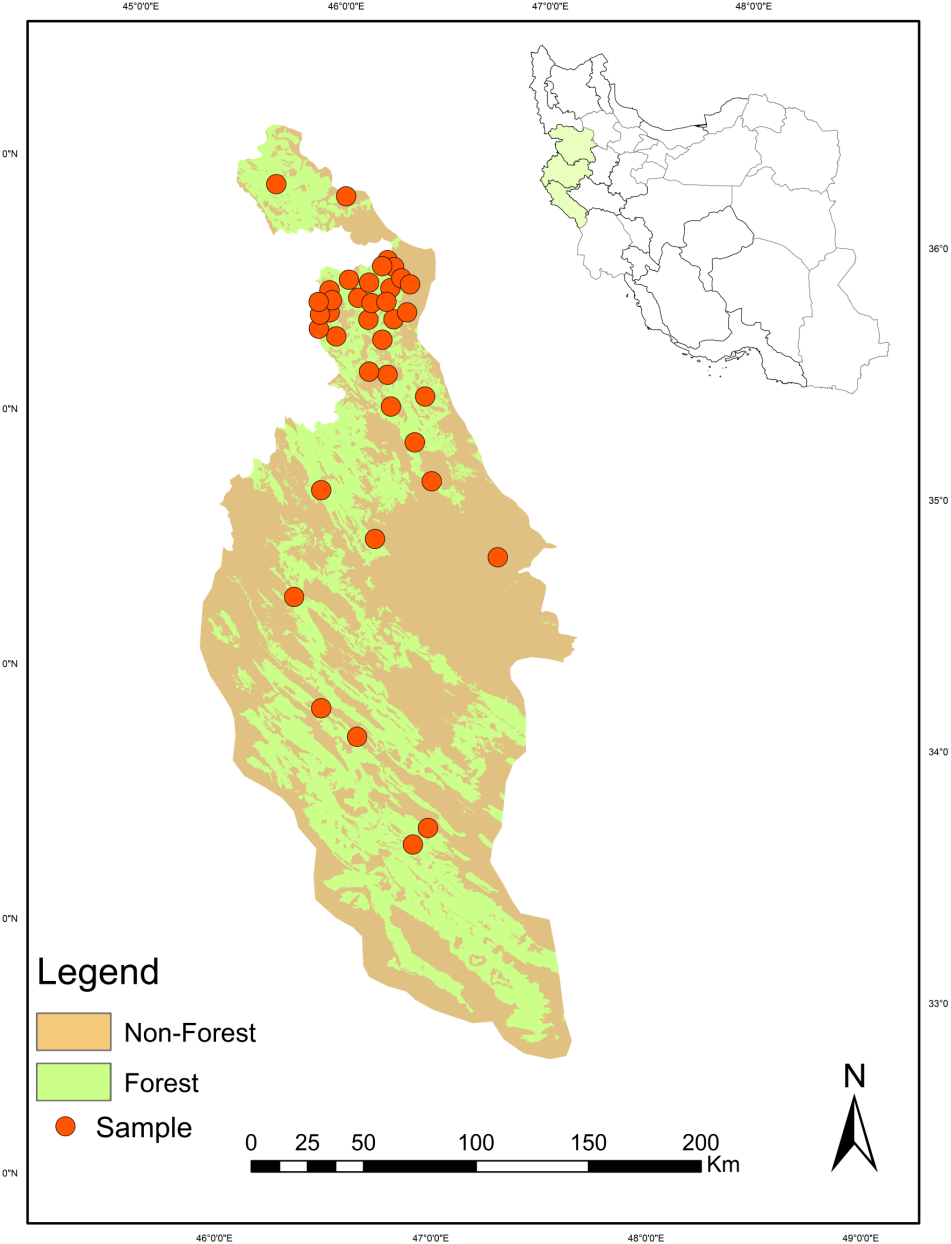
Location of the study area and sampling points in the Zagros forest in western Iran. Each red dot represents the sampling location of an individual Persian squirrel.

Individuals of the Persian squirrel were sampled in the north of the Zagros Forest in Iran (2016–2018) (Kurdistan, Kermanshah and Ilam; Figure 1). Tissues including small pieces of fur, skin, muscle, and ear were sampled from live, captured animals or corpses, and the geographical coordinates of sampling points were recorded. For sampling of live squirrels, conducted in cooperation with the Department of Environment in Iran, live Persian squirrels illegally taken by hunters or local people were sampled, and all live squirrels were released after capture (Asadi Aghbolaghi et al., 2019). We only included in our dataset samples from animals whose location of capture was certain. Tissues were preserved in ethanol at −20°C. No samples from zoos were considered in this research because of the risk of hybridization in captivity (Taberlet et al., 1999; Figure 1). A total of 38 individuals were included in this study, all identified previously as belonging to the northern Zagros population group based on mitochondrial and nuclear gene sequences (population group G4 in Asadi Aghbolaghi et al., 2019).

### 2.2 DNA extraction and microsatellite amplification

DNA was extracted from tissue samples using a DNeasy Blood and Tissue Kit (Qiagen), with a final elution volume of 50 μL. We amplified 19 dinucleotide microsatellite loci (Scv1, Scv3, Scv4, Scv6, Scv8, Scv9, Scv12, Scv13, Scv14, Scv15, Scv16, Scv18, Scv19, Scv20, Scv23, Scv24, Scv25, Scv27, Scv31, Scv32; Hale, Lurz, et al., 2001). Microsatellite loci were amplified individually in 15 μL reactions containing 0.5 μL template DNA, 1 × AmpliTaq buffer, 1.5 mM MgCl_2_, 0.75 mM each dNTP, 0.5 mM each primer, and 0.2 U AmpliTaq DNA polymerase (Applied Biosystems) under the following cycling conditions: 95°C for 2 min; 35 cycles of 94°C for 15 s, annealing temperature (48, 52, or 54°C) for 15 s (see Hale, Lurz, et al., 2001), and 72°C for 15 s; followed by 72 °C for 10 min. PCR amplifications were performed in a PTC 0200 DNA Engine Cycler (Bio‐Rad). Forward primers were labeled with a fluorescent dye and PCR products were visualized and sized on an Applied Biosystems 3730S capillary DNA analyzer, under standard run conditions, with 500 LIZ as the internal size standard. Electropherograms generated by the DNA analyzer were scored using GeneMarker software (v.2.6.3) and all genotypes were checked manually (Hulce et al., 2011).

### 2.3 Population genetic analysis

Since all of our samples belonged to a single genetic group, as determined by previous mitochondrial and nuclear gene analyses (Asadi Aghbolaghi et al., 2019), and we did not sample spatially distinct populations, all basic population genetic analyses were performed treating all samples as a single population. Micro‐checker software was used to check scoring errors, stutter, or null alleles (Van Oosterhout et al., 2004). Gametic disequilibrium between all pairs of microsatellite loci was tested using POPGENE (Version: 1.32; Panahabadi et al., 2021, 2022; Yeh Francis et al., 1999). Null alleles and deviations from Hardy–Weinberg equilibrium for each locus were estimated using Genepop (Version 4.2.1; Panhabadi et al., 2022; Rousset, 2008). Unbiased gene diversity per locus and inbreeding coefficient (*F*_IS_) were estimated using FSTAT (Version 1.2; Goudet, 1995; v.4.2), with the Bonferroni correction provided by FSTAT to infer significant values. The number of alleles, observed heterozygosity and expected heterozygosity were estimated using GeneAlex (v.6.5; Peakall & Smouse, 2006) and Arlequin (version 3.5.2.2; Excoffier & Lischer, 2010). Finally, the genetic distance between each pair of individuals was estimated using Microsatellite Analyzer (MSA) 4.05 (Dieringer & Schlötterer, 2003) based on the proportion of shared alleles (Dps‐1n; Bowcock et al., 1994); these genetic distances were also used as the response variable in our IBD and IBR analyses (see below). In simulations assessing the performance of individual‐based genetic distances in landscape genetic tests, Shirk et al. (2017) found that Dps performed well, providing high accuracy while making no biological assumptions.

We tested for the presence of genetic structure or subpopulations within our sample using Bayesian clustering implemented in the program STRUCTURE v.2.3.4 (Pritchard et al., 2000). We used an admixture model with correlated allele frequencies, and burn‐in and run length set to 100,000 and 1,000,000 runs, respectively. We set the number of clusters (K) from 1 to 4, and executed 10 independent runs of the MCMC algorithm for each value of *K*. Output from all runs was compiled using STRUCTURE HARVESTER v. 0.6.94 (Earl & VonHoldt, 2012) and admixture assignments of individuals plotted with the program DISTRUCT v. 1.1 (Rosenberg, 2004). The most likely value of K was determined based on the highest posterior probability of the data given K (Pritchard et al., 2000) as well as the highest rate of change in K (Delta K; Evanno et al., 2005).

### 2.4 Isolation by resistance and distance

**FIGURE 2:**
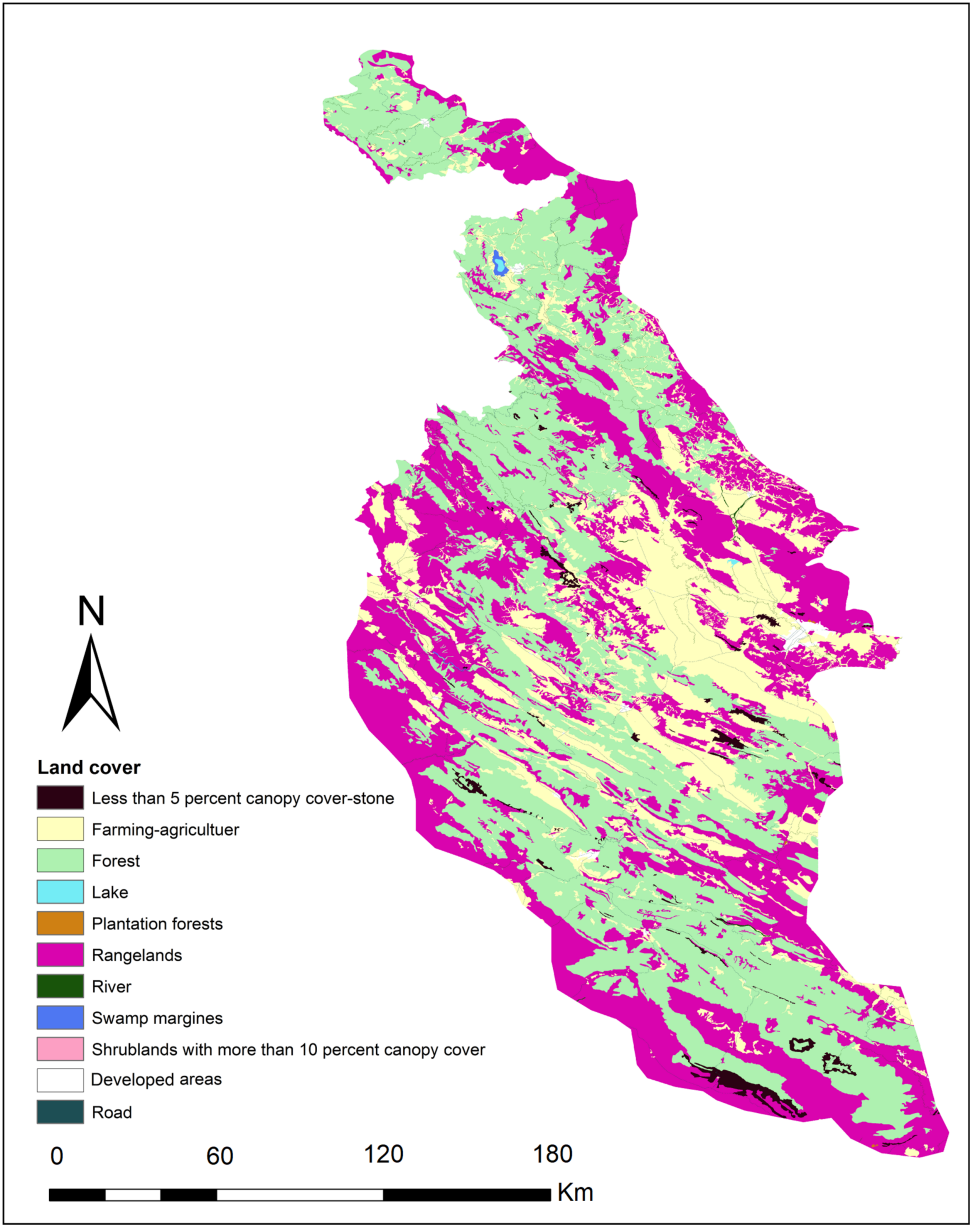
Land cover map with 11 classes, produced by Forests, Range and Watershed Management Organization in Iran, 2017.

We created a composite land cover map (produced by Forests, Range and Watershed Management Organization in Iran, 2017), in ArcGIS (v.10.3; Figure 2) with nine initial thematic classes: developed areas, farming and agriculture, forests, lakes, plantation forests, rangelands, shrublands with more than 10% canopy cover, lands with stone surface and <5% canopy cover, and swamp margins. Then we added a road layer and river layer to the land cover map to test hypotheses about the restrictive effects of roads and rivers on gene flow (Figure 2). The geographical coordinates of sampling points (i.e., individual squirrel samples) and all land cover and road layers were mapped based on the WGS_1984 coordinate system, and the universal transverse mercator (UTM) projection. We aggregated the surface to 300*300 m resolution, because the extent of our study area made finer resolutions not tractable for optimization; for example, at 30 × 30 m resolution our surface had more than 80 million cells, while most successful optimization analyses have <2 million raster cells (Peterman, 2018). Furthermore, given mean and maximum observed natal dispersal distances of ~1041 m and >4000 m, respectively, in closely related Eurasian red squirrels (Wauters et al., 2010), a 300 m resolution should be appropriate to capture landscape effects on gene flow and genetic structure.

We evaluated landscape resistance using resistance distances, which can capture multiple potential routes of movement through the landscape and do not assume that the organism has perfect knowledge of the landscape (McRae, 2006), and an individual‐based analysis framework (Laurence et al., 2013; Seaborn et al., 2019). We used the R package ResistanceGA (v. 4.1–16, Peterman, 2018), which uses a genetic algorithm to search parameter space for the best‐fit resistance surface parameterization, as well as the functional form of the relationships between landscape covariates and the surface (Bauder, Cervantes, et al., 2021; Bauder, Peterman, et al., 2021; Darinot et al., 2021). In ResistanceGA, for each evaluated parameterization of the resistance surface, a nonlinear functional transformation is applied, pairwise resistance distances between sampling points are estimated, and these are fit to genetic distances using a linear mixed‐effects model with a maximum likelihood population effects (MLPE) structure to account for the pairwise nature of the variables (Clarke et al., 2002). The best‐fit parameterization of the resistance surface is then selected based on Akaike's Information Criterion adjusted for small‐sample sizes (AICc, Burnham & Anderson, 2004), and marginal $R_{m}^{2}$ and conditional $R_{c}^{2}$ (i.e., the proportion of variance explained by fixed‐effect factors only, and both fixed and random‐effect factors, respectively) are also provided (Johnson et al., 2014; Nakagawa & Schielzeth, 2013; Row et al., 2017). Resistance distances used in ResistanceGA were estimated using CIRCUITSCAPE (version 5.0.0; Kivimaki et al., 2014; McRae, 2006; McRae et al., 2008) written in the Julia programming language (version 0.6.4; https://julialang.org/, accessed 1 Aug 2018). In ResistanceGA, we used the GA.prep function, setting max.cat = 3000 and maxiter = 100. ResistanceGA also evaluates a model of IBD (equivalent to a surface with resistance of all pixels set to 1) and a null model with only the intercept and random effects. We compared empirical support for the best‐fit parameterization of the resistance surface, the IBD model, and the null model using AICc scores generated by ResistanceGA, interpreting models within two AIC units (ΔAICc ≤2) of the best‐supported model as being well supported (Row et al., 2017; Russo et al., 2016).

Because of the central role of forest in our hypothesis of how landscape influences movement and gene flow in the Persian squirrel, we conducted a complementary analysis, also individual‐based, to clarify and focus on the effect of forested land cover on the genetic structure of the species. The land cover map was converted to a binary surface with two classes: Forest and Non‐Forest. In some cases, analysis of binary habitat‐non habitat surfaces can provide complementary or novel insights compared with the analysis of multi‐variable surfaces (van Rees et al., 2018; Van Strien et al., 2012). Resistance distances between sampling points were estimated using CIRCUITSCAPE (v. 3.5.2; McRae, 2006) with resistance of Forest set to 1 and that of non‐Forest set to a range of different values: 5, 10, 50, 100, and 500. We also tested a parameterization representing the reverse of our hypothesis that forests facilitate movement and gene flow, in which the resistance of non‐Forest was set to 1 and that of Forest to 500 (i.e., forested areas were represented as having very low permeability). Genetic distances between samples were then fit to the resistance distances generated from each resistance surface parameterization, and were also fit separately to pairwise Euclidean distance between populations (representing IBD), using MLPE models; specifically, we fit mixed models with maximum likelihood using the R package nlme (Pinheiro et al., 2020), and using a correlation structure specified by corMLPE (Pope, 2020) to account for the pairwise structure of the data (Clarke et al., 2002), following code provided at (https://github.com/nspope/corMLPE).

## 3 RESULTS

### 3.1 Population genetic analysis

Of 19 microsatellite loci, we found three monomorphic and 16 polymorphic loci in our sample; only the polymorphic loci were retained for analysis. None of the 16 polymorphic loci showed significant evidence of null alleles, and linkage disequilibrium and deviations from Hardy–Weinberg equilibrium were not detected after Bonferroni correction. The total observed number of alleles per locus among these polymorphic loci varied from 2 to 12. The overall mean expected heterozygosity (*H*_E_) per locus was 0.489 and the mean inbreeding coefficient (*F*_IS_) was 0.149 (*p* < .01; Table 1). Two loci, Scv4 and Scv20, had *F*_IS_ = 1.0; these extreme *F*_IS_ values occurred because the loci were largely monomorphic, except in two samples (ES‐1175 and ES‐1176) that were homozygous for rare alleles at both loci. Both samples are from the southern end of our sampling area, in a contact zone between the northern Zagros and southern Zagros populations of this species (See Asadi Aghbolaghi et al., 2019). These individuals may carry these rare alleles as a result of mixed ancestry from the southern Zagros population. Excluding loci Scv4 and Scv20 reduced *F*_IS_ to 0.131.

**TABLE 1:** Summary statistics for 19 microsatellite loci in the Persian squirrel from North Zagros, Iran: number of alleles, observed heterozygosity (*H*_O_), expected heterozygosity (*H*_E_) and inbreeding coefficient (*F*_IS_).

Locus	Size range (bp)	Alleles	*H*_O_	*H*_E_	*F*_IS_
Scv1	164–160	5	0.421	0.523	0.197
Scv3	255–241	7	0.631	0.745	0.156
Scv4	220–214	3	0	0.102	1.000
Scv6	214–202	8	0.894	0.779	−0.150
Scv8	204–194	5	0.486	0.620	0.219
Scv9	197–193	3	0.578	0.580	0.003
Scv12	211–199	3	0.052	0.052	−0.007
Scv14	218–196	8	0.394	0.482	0.183
Scv15	182	Monomorphic	–	–	NA
Scv16	204–198	4	0.277	0.571	0.518
Scv18	259–251	6	0.457	0.559	0.186
Scv19	207–203	4	0.315	0.430	0.269
Scv20	168–166	2	0	0.051	1.000
Scv23	187–179	5	0.631	0.691	0.087
Scv24	170–156	12	0.710	0.884	0.199
Scv25	181–161	5	0.157	0.198	0.206
Scv27	159	Monomorphic	–	–	NA
Scv31	160	Monomorphic	–	–	NA
Scv32	238–226	7	0.676	0.563	−0.204
Total			0.417	0.489	0.149

**FIGURE 3:**
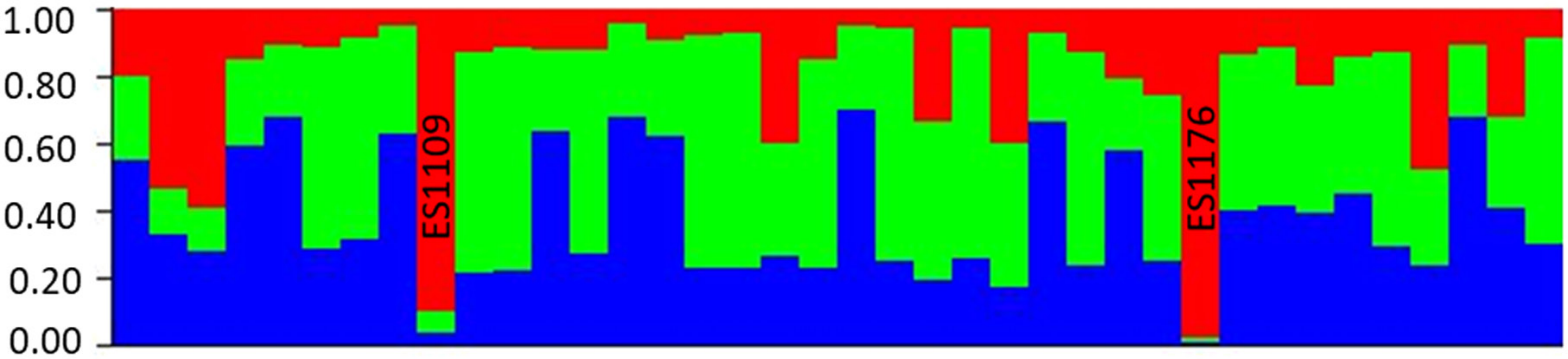
Analysis using STRUCTURE showed that the most probable number of genetic clusters in our sample (*K*) is three. Admixture assignments for individual squirrels (each represented by a vertical bar) revealed that all individuals have a mix of ancestry from all three clusters and did not indicate any distinct subpopulations within our sample. Only two individuals showed more than 80% assignment to any one of the three clusters: ES‐1109 and ES‐1176 showed approximately 90% and 97% assignment, respectively, to the same cluster.

Analysis using STRUCTURE indicated that the most likely number of genetic clusters in our sample (*K*), based on the posterior probability of the data given *K*, was three (Mean estimated ln probability of the data ± SD = −1157 ± 3.21). The next most likely number of clusters, with similar posterior probability and standard deviation was one (Mean est. ln *p* of data ± SD = −1182 ± 0.92). Based on the rate of change in *K* (Evanno et al., 2005), the most likely number of clusters was also three (this method cannot test for *K* = 1). However, individual admixture assignments showed that all individuals have a mix of ancestry from all three clusters and did not indicate any distinct subpopulations within our sample (Figure 3). The mean level of assignment to each of the three different clusters, across all individuals and across 10 runs at *K* = 3, was approximately 40%, 38%, and 22%. Only two individuals showed more than 80% assignment to any one of the three clusters: ES‐1109 and ES‐1176 showed approximately 90% and 97% assignment, respectively, to the same cluster.

### 3.2 Isolation by distance and resistance

**FIGURE 4:**
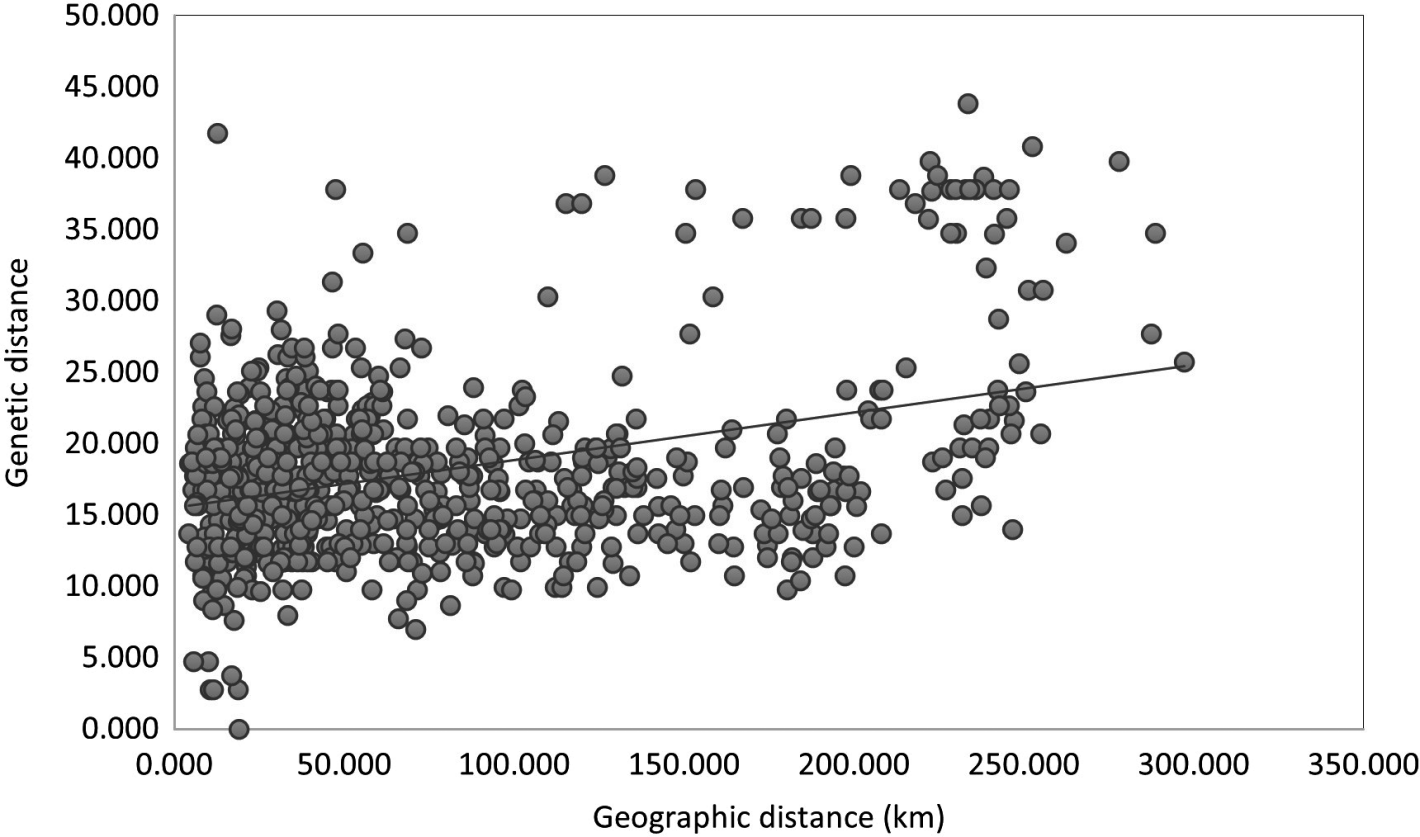
Positive correlation between individual‐based genetic distance and geographical distance in the Persian squirrel in the North Zagros.

**FIGURE 5:**
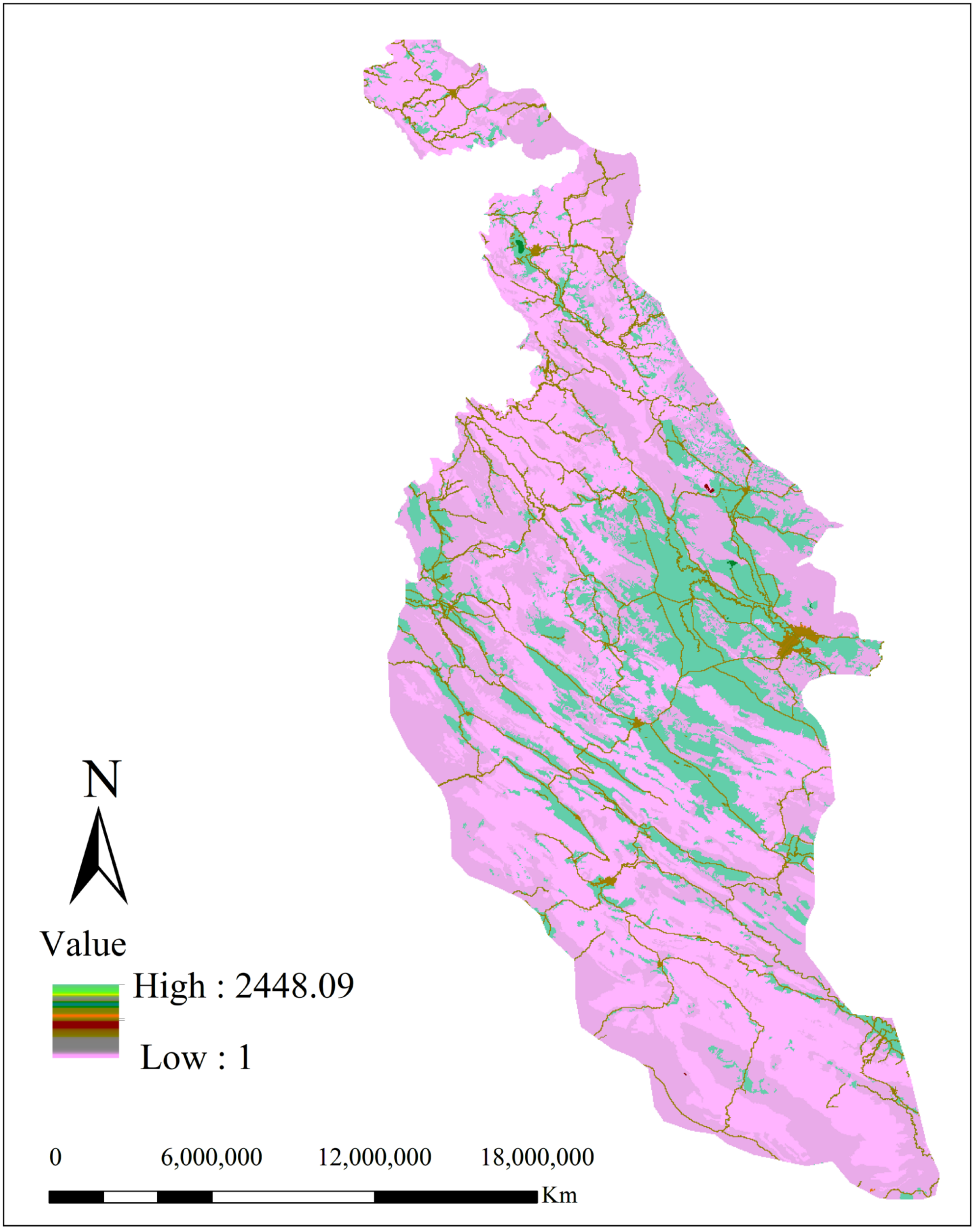
Top‐ranked optimized landscape resistance parameterization for the Persian squirrel in the North Zagros area. The lowest resistance is assigned to lands with stone surface and <5% canopy cover (feature 1), forest (feature 3), and rangelands (feature 6). Moderate resistance is associated with shrublands with more than 10% canopy (feature 9), developed areas (feature 10), and plantation forest (feature 5). Highest resistance is associated with swamp margins (feature 8), farming‐agriculture (feature 2), lakes (feature 4), rivers (feature 7), and roads (feature 11).

In analyses conducted using ResistanceGA, the distance‐only model outperformed both the null and landscape resistance models in explaining individual‐based genetic distance, indicating support for isolation by distance (IBD) in the Persian squirrel in the Zagros region (Table 2a, Figure 4). In the best landscape resistance model identified, the lowest resistances were associated with areas with stone surface and less than 5% canopy cover (feature 1), forest cover (feature 3), and rangelands (feature 6) while the highest resistance was associated with swamp margins (feature 8) and farming‐agriculture (feature 2) (Table 2b, Figure 5). However, the landscape resistance model performed considerably more poorly than the null model, therefore there is overall little evidence that contemporary land cover is affecting patterns of genetic differentiation in the Persian squirrel in our study area.

**TABLE 2:** (a) Comparison of the best landscape resistance surface, as parameterized by ResistanceGA, IBD, and null models for explaining individual‐based genetic distances in the Persian squirrel in the North Zagros. Models were compared based on the corrected Akaike information criterion (AICc), *K* is the number of model parameters, $R_{m}^{2}$ is the marginal *r*‐square and $R_{c}^{2}$ is the conditional *r*‐square. (b) Resistance values associated with the best landscape resistance parameterization. All outputs were determined using ResistanceGA.

(a)						
Surface	Log‐likelihood	*K*	ΔAICc	AICc	$R_{m}^{2}$	$R_{c}^{2}$
Distance only	428.93	2	0	−853.51	0.026	0.68
Null	423.23	1	9.15	−844.36	0	0.68
Resistance	429.75	12	30.49	−823.02	0.027	0.68

(b)										
Feature 1 (Less than 5% canopy; stone surface)	Feature 2 (Farming‐agriculture)	Feature 3 (Forest)	Feature 4 (Lake)	Feature 5 (Plantation forest)	Feature 6 (Rangelands)	Feature 7 (River)	Feature 8 (Swamp margins)	Feature 9 (Shrublands with more than 10% canopy)	Feature 10 (Developed areas)	Feature 11 (Roads)
1	2425.2	46.1	1829.1	1424.3	59.3	1489.0	2448.1	1032.3	1295.8	1455.7

**FIGURE 6:**
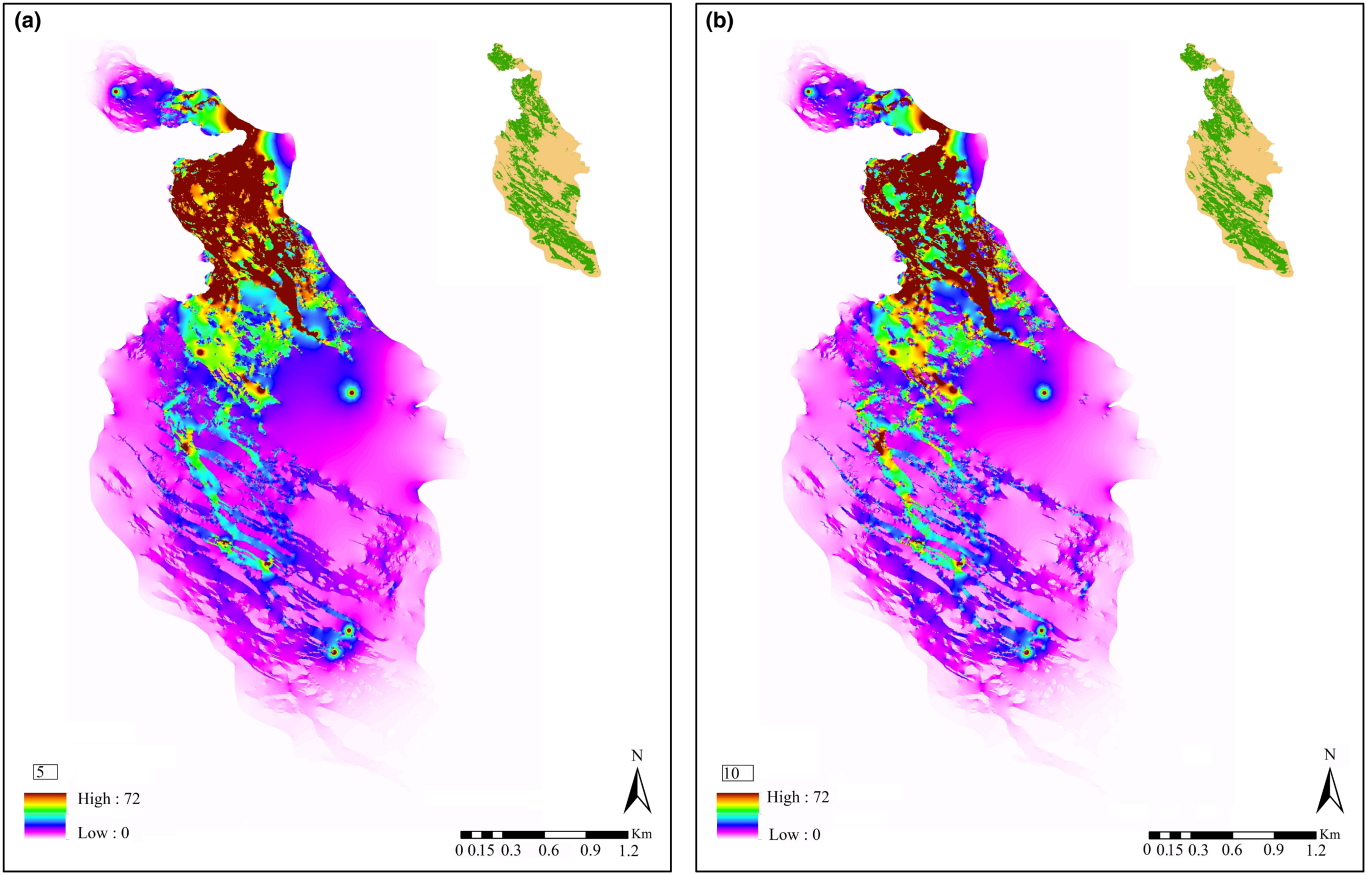
Maps of land cover (inset; green = forest and beige = non‐forest) and estimated current for the Persian squirrel in the North Zagros, based on the best‐fit binary surface with the resistance of non‐forest set to 5 (a) and 10 (b). Areas that are pale pink and whitish have low current and high resistance and regions that are brownish and yellow have high current and low resistance.

Our binary analysis of the effects of forest versus non‐forest on the genetic structure of the Persian squirrel showed highest support for models in which non‐forested areas have resistances of 5 and 10, although the IBD model was equally well supported (Tables 3 and 4). With increasing resistance of non‐forest beyond 10, the resulting binary surfaces performed increasingly poorly in explaining genetic distance (Table 4, Figure 6). The reverse landscape resistance surface with higher resistance of forest compared with non‐forest performed the most poorly, with the highest AICc (Table 4).

**TABLE 3:** Relationship between individual‐based genetic distance and geographical distance (IBD) in the Persian squirrel in the North Zagros, based on maximum likelihood fit of a MLPE model.

Correlation	Coefficient ± SE	ΔAICc	AICc	*p*‐value
0.356	0.025 ± 0.0076	0.55	−849.33	.001***

*Note*: ΔAICc is relative to the best‐fit model for landscape resistance based on a binary forested versus non‐forested surface (Table 4). The significance level is denoted by asterisks (***) (Table 4).

**TABLE 4:** Comparison of maximum likelihood fits of landscape resistance models explaining individual‐based genetic distance in the Persian squirrel, based on binary resistance surfaces.

Resistance of non‐forest	AICc	ΔAICc	*p*‐value
5	−849.88	0	.0006***
10	−849.56	0.32	.0006***
50	−842.60	7.28	.041
100	−841.70	8.18	.071
500	−840.76	9.12	.128
Reverse with forest resistance of 500	−839.06	10.82	.433
IBD model	−849.33	0.55	.001***

*Note*: Resistance distances were derived with CIRCUITSCAPE (v. 3.5.2), using parameterizations of the resistance surface in which forested areas were assigned resistance of 1 and all non‐forested areas were assigned resistance ranging from 5 to 500. A reversed resistance surface parameterization in which non‐forested areas had resistance of 1 and forested areas had resistance of 500 was also tested. The isolation by distance (IBD) model is included for comparison (full model parameters provided in Table 3). The significance level is denoted by asterisks (***).

To ensure our results were not being driven by the two individuals that were most genetically distinct in our sample, both identified by STRUCTURE as having high admixture ancestry from a single cluster (ES‐1109 and ES‐1176), we re‐ran our analyses with these two samples removed. Excluding these samples did not alter our main conclusions; in particular, the Distance only model was still the best‐supported model in the ResistanceGA analysis (ΔAICc = 9.21 for Null model and ΔAICc = 31.39 for Resistance model), and the correlation between pairwise genetic distance and Euclidean distance was weaker but still significant (*β* ± SE = 0.00025 ± 0.0001, *p* = .036, *r* = .076).

## 4 DISCUSSION

This is the first landscape genetics study for the Persian squirrel; we focused on analysis of IBD and IBR using an individual‐based approach, to identify features that may potentially explain contemporary genetic structure in the northern Zagros region. Microsatellite genotyping of samples from this region showed a moderate amount of genetic diversity (*H*_O_: 0.417, *H*_E_: 0.489), similar to that observed in other tree squirrels, including the Delmarva fox squirrel (*S. niger cinereus*; Lance et al., 2003) and Eurasian red squirrel (*S. vulgaris*; Hale, Bevan, & Wolff, 2001; Trizio et al., 2005). We observed a significant and relatively high level of inbreeding (*F*_IS_: 0.149), which is also consistent with inbreeding levels observed in at least some populations of the Eurasian red squirrel using microsatellites (Hale, Bevan, & Wolff, 2001, Trizio et al., 2005). High inbreeding estimates in these other studies have been attributed to the possibility of sampling across populations or subpopulations (Hale, Bevan, & Wolff, 2001, Trizio et al., 2005), and in our case may potentially be attributed to some genotypes showing admixture or introgression from the southern Zagros genetic group.

Bayesian analysis of clustering using STRUCTURE (Pritchard et al., 2000) identified three as the most likely value of *K*. However, the sampled individuals did not group into distinct subpopulations; instead, all individuals were inferred to share some degree of admixture from all three genetic clusters. This suggests that our samples belong to a single contemporary population or subpopulation, which may have experienced past admixture among ancestrally separated lineages. During the Pleistocene, this species experienced cycles of refugial isolation and subsequent expansion in this region; a relatively recent history of isolation among distinct genetic populations followed by intermixing (Asadi Aghbolaghi et al., 2019) could explain the signal of admixture across all individuals in our sample. We cannot ascertain how, or if, the three genetic clusters revealed here correspond to the five distinct sub‐specific groups of the Persian squirrel identified based on mitochondrial and nuclear gene sequences (Asadi Aghbolaghi et al., 2019). An expanded analysis that includes a reasonable number of samples from each of the different groups would be needed to determine this. One of the two individuals showing very high admixture from one of the clusters (ES‐1176) does occur in the region of overlap between the North and South Zagros groups; however, the second individual (ES‐1109) was sampled further north.

Our results suggested geographical distance as an important factor that structures contemporary genetic variation among Persian squirrels in the North Zagros. We found a significant pattern of IBD among the samples in our study area, but little evidence for an effect of landscape heterogeneity, particularly forest distribution, on genetic structure and gene flow. There was no support for IBR in our analyses using ResistanceGA, where the potential effect of multiple land covers was assessed simultaneously. An alternative analysis based on a simple binary classification of the landscape revealed only a weak role for forest in facilitating gene flow and dispersal. Therefore, our analyses suggest spatial separation may be the main factor that limits gene flow and dispersal of the Persian squirrel in this area (Bauder, Cervantes, et al., 2021). In many mammal species, IBR models that account for the heterogeneity in species' distributions and migration rates better explain the genetic structure of populations in comparison to IBD models (Coulon et al., 2006; Geiser et al., 2013; Klug & Wisely, 2011; McRae, 2006; Mullins et al., 2014). Nonetheless, there are several other mammals, including some with high dispersal ability like Goitered gazelle, *Gazella subgutturosa*, or white‐tailed deer, *Odocoileus virginianus*, for which distributions are better explained by geographical distance than landscape matrix (Bauder, Cervantes, et al., 2021; Khosravi et al., 2018; Mullins et al., 2014).

The resistance surface parameterization that best fit our genetic data, as determined by the ResistanceGA optimization process, had lowest resistances associated with forest, but also rocky areas with low canopy cover, and rangelands. This suggests that perhaps high canopy cover may not be strictly necessary to facilitate movement of squirrels across the landscape, but that other natural or semi‐natural areas with a low level of development may also be suitable. It is not unusual for animals to cross areas that are unsuitable as habitat, as long as their movement is not impaired or mortality risk is not elevated; for example, kinkajous, *Potos flavus*, can disperse readily through farms and pastures even though they are also arboreal and highly reliant on forests as habitat (Keeley et al., 2017). Highest resistances were associated with agriculture, as well as water bodies and swamp margins. This suggests squirrels may avoid areas of higher human activity, as well as wet areas subject to flooding. Consistent with these inferences, developed areas, roads, lakes, and rivers also had relatively high resistances. However, even though this was the resistance surface parameterization that best fit genetic distances, it was considerably less well supported than the IBD model and was also less supported than a null model. Therefore, these resistance values should not be over‐interpreted and can only suggest possible hypotheses that could be tested in future studies with larger sample sizes and perhaps better sampling across different land cover types. Another caveat about these analyses is that some of the land covers, including those with among the lowest and highest resistance in the best‐supported surface (e.g., stone‐covered areas with <5% canopy cover, and swamp margins), occurred at very low frequency. For example, stone‐covered areas with <5% canopy cover occurred in only ~0.57% of our study area, compared with total forest cover of 18.41%. The relative abundance of different land covers in a study area is known to influence the results of landscape genetic analyses, and the power to detect significant effects of particularly under‐ or over‐represented land cover types (Short Bull et al., 2011). This may explain why our analysis based on a binary classification of the landscape, in which the two land cover categories of forest and non‐forest occur at more similar frequency, was able to reveal some role, albeit weakly, for forest in facilitating gene flow and dispersal. Here, the resistance surfaces that best fit genetic distance were those with the lower resistance values of 5 and 10 assigned to non‐forest; however, a model of IBD was almost just as well supported. Similar landscape genetic studies of the Persian squirrel in other parts of its range, and in landscapes with different overall landscape composition, will be important in determining the extent to which the weak landscape effects detected here may be an artifact of our study area, or reflect the real effects of land cover on squirrel movement.

We used an individual‐based approach to assess IBD and IBR. Individual‐based sampling and analysis are widely used in landscape genetics, particularly for organisms that do not show discrete subpopulation structure or are continuously distributed across space (Bauder, Anderson, et al., 2021; Draheim et al., 2018; Laurence et al., 2013). However, individual‐based analyses can sometimes lead to different conclusions about landscape effects on genetic structure and gene flow compared to population‐based analyses. For example, Seaborn et al. (2019) found that although individual‐based sampling was helpful for increasing sampling extent and density, it may lack power for model comparisons compared with population‐based sampling. Kunz et al. (2022) also found differing support for IBD versus IBR among individuals compared to among subpopulations. In our case, given the lack of clear subpopulations or distinct sampling areas within our sample, an individual‐based approach was both necessary and justified. However, along with the small sample size, this approach may have limited our power to detect significant IBR.

Our inability to detect stronger evidence for IBR may also be a function of time lags. Lags in species' responses to environmental change can lead to a mismatch between patterns of genetic variation and landscape structure (Anderson et al., 2010; Du Toit et al., 2016; Epps & Keyghobadi, 2015). The Zagros forests have suffered dramatic declines in recent decades, due to climatic change and different forms of traditional land use (Khalyani & Mayer, 2013; Sepahvand et al., 2021). The landscape here has changed quickly and recently, and it is possible that the genetic structure of the Persian squirrel is lagging, obscuring an association between contemporary landscape structure and patterns of genetic differentiation. Although use of individual‐based genetic distances should facilitate detection of contemporary movement, it would not eliminate genetic time lags. Furthermore, the Persian squirrel generally has large populations, with a density of around 0.2 individuals per hectare (Amr et al., 2006; Zevgolis et al., 2022), and a moderately long lifespan (~15 years; Karami et al., 2016); in addition, the closely related Eurasian red squirrel demonstrates moderate dispersal, with ~75% of individuals estimated to emigrate from the natal home range with a mean dispersal distance of 1014 ± 925 m (Wauters et al., 2010). Large populations, long lifespan, and moderate dispersal all contribute to a relatively slow response of genetic structure to landscape change (Epps & Keyghobadi, 2015). Again, studies in other regions with a different history of landscape change may shed light on this issue. Our previous work found that Persian squirrels in the South Zagros were part of a different population group than those in the North Zagros (Asadi Aghbolaghi et al., 2019); further sampling and landscape genetic work in that area will be particularly important to understand fully the factors affecting Persian squirrel genetic variation, and contemporary population structure and movement, in western Iran.

Even if forest cover has only a weak influence on movement of the Persian squirrel, as the unique source of their resources forest is still critically important for persistence of the species. Forests, and the tree resources they contain, are necessary for supporting reproduction and survival, and for the maintenance of squirrel population numbers (Khalili et al., 2016). Therefore, it is important to maintain forest on the landscape to support squirrel populations. Our results suggest that squirrels may potentially be able to move through other land cover types to access these necessary resources, including areas with low canopy cover as long as they not too wet or too highly developed. Given the role of IBD that we identified, our results further suggest that the nature of the intervening matrix may be less important than simply ensuring that patches of forest and tree resources are not too spatially isolated. Therefore, from a landscape management perspective, all patches of forest and tree cover on the landscape can be beneficial for Persian squirrels, even if they are interspersed by other land cover types. Management focused on maintaining and increasing forest cover and appropriate tree species on the landscape, regardless of the matrix, will be beneficial for Persian squirrels by both adding habitat and resources, and facilitating movement by decreasing distances among resource patches.

Landscape genetic analyses provide a potentially powerful, empirical means to understand how landscape factors and landscape change influence gene flow and movement, thereby informing conservation actions. Based on analysis of a relatively small number of samples, we did not identify significant effects of land cover on genetic differentiation of the Persian squirrel. Instead, our current research suggests that geographical distance is the main factor that has shaped the genetic structure and gene flow of this species in the North Zagros. Our results point to the importance of maintaining and increasing forests on the landscape and to decreasing spatial isolation of habitat patches. Future work should provide more samples from this region and across the species' range to identify possible populations and subpopulations, patterns of ancestry admixture, and explore landscape genetics of the Persian squirrel in other areas, particularly in the South Zagros. This would give us a more complete picture of how landscape composition and configuration influence the movements and genetic structure of Persian squirrel throughout the Zagros forests, and across its range.

## AUTHOR CONTRIBUTIONS

**Marzieh Asadi Aghbolaghi:** Conceptualization (lead); data curation (lead); formal analysis (lead); funding acquisition (lead); investigation (lead); methodology (lead); project administration (lead); resources (lead); software (lead); supervision (lead); validation (lead); visualization (lead); writing – original draft (lead); writing – review and editing (lead). **Nusha Keyghobadi:** Conceptualization (lead); formal analysis (supporting); investigation (supporting); methodology (lead); resources (supporting); software (lead); supervision (lead); validation (lead); visualization (lead); writing – original draft (supporting); writing – review and editing (lead). **Zeinab Azarakhsh:** Formal analysis (supporting); software (supporting). **Marzieh Dadizadeh:** Formal analysis (supporting); software (supporting). **Shahab Asadi Aghbolaghi:** Methodology (supporting). **Navid Zamani:** Methodology (supporting).

## FUNDING INFORMATION

This study was conducted without access to institutional funding option.

## Data Availability

Microsatellite genotypes and sampling locations are located in The Dryad Data Platform repository, https://doi.org/10.5061/dryad.p8cz8w9wb.
